# The Effect of KSK-94, a Dual Histamine H_3_ and Sigma-2 Receptor Ligand, on Adipose Tissue in a Rat Model of Developing Obesity

**DOI:** 10.3390/ph17070858

**Published:** 2024-07-01

**Authors:** Magdalena Kotańska, Monika Zadrożna, Monika Kubacka, Kamil Mika, Katarzyna Szczepańska, Barbara Nowak, Alessio Alesci, Anthea Miller, Eugenia Rita Lauriano, Katarzyna Kieć-Kononowicz

**Affiliations:** 1 Department of Pharmacological Screening, Jagiellonian University Medical College, Medyczna 9, PL 30-688 Kraków, Poland; kamil1991.mika@gmail.com; 2 Department of Cytobiology, Jagiellonian University Medical College, Medyczna 9, PL 30-688 Kraków, Poland; monika.zadrozna@uj.edu.pl (M.Z.); barbara.anna.nowak@uj.edu.pl (B.N.); 3 Department of Pharmacodynamics, Jagiellonian University Medical College, Medyczna 9, PL 30-688 Kraków, Poland; monika.kubacka@uj.edu.pl; 4 Department of Medicinal Chemistry, Maj Institute of Pharmacology, Polish Academy of Sciences, Smętna 12, PL 31-343 Kraków, Poland; szczepanskatarzyna@gmail.com; 5 Department of Chemical, Biological, Pharmaceutical and Environmental Sciences, University of Messina, Viale F. Stagno d’Alcontres 31, I-98166 Messina, Italy; alessio.alesci@unime.it (A.A.); elauriano@unime.it (E.R.L.); 6 Department of Veterinary Sciences, University of Messina, I-98168 Messina, Italy; anthea.miller@studenti.unime.it; 7 Department of Technology and Biotechnology of Drugs, Faculty of Pharmacy, Jagiellonian University Medical College, Medyczna 9, PL 30-688 Kraków, Poland; katarzyna.kiec-kononowicz@uj.edu.pl

**Keywords:** developing obesity, beige adipocyte, white adipose tissue browning, dual histamine H_3_ and sigma-2 receptor antagonist

## Abstract

Background: Numerous studies highlight the critical role that neural histamine plays in feeding behavior, which is controlled by central histamine H_3_ and H_1_ receptors. This is the fundamental motivation for the increased interest in creating histamine H_3_ receptor antagonists as anti-obesity medications. On the other hand, multiple other neurotransmitter systems have been identified as pharmacotherapeutic targets for obesity, including sigma-2 receptor systems. Interestingly, in our previous studies in the rat excessive eating model, we demonstrated a significant reduction in the development of obesity using dual histamine H_3_/sigma-2 receptor ligands. Moreover, we showed that compound KSK-94 (structural analog of Abbott’s A-331440) reduced the number of calories consumed, and thus acted as an anorectic compound. Therefore, in this study, we extended the previous research and studied the influence of KSK-94 on adipose tissue collected from animals from our previous experiment. Methods: Visceral adipose tissue was collected from four groups of rats (standard diet + vehicle, palatable diet + vehicle, palatable diet + KSK-94, and palatable diet + bupropion/naltrexone) and subjected to biochemical, histopathological, and immunohistochemical studies. Results: The obtained results clearly indicate that compound KSK-94 prevented the hypertrophy and inflammation of visceral adipose tissue, normalized the levels of leptin, resistin and saved the total reduction capacity of adipose tissue, being more effective than bupropion/naltrexon in these aspects. Moreover, KSK-94 may induce browning of visceral white adipose tissue. Conclusion: Our study suggests that dual compounds with a receptor profile like KSK-94, i.e., targeting histamine H_3_ receptor and, to a lesser extent, sigma-2 receptor, could be attractive therapeutic options for patients at risk of developing obesity or with obesity and some metabolic disorders. However, more studies are required to determine its safety profile and the exact mechanism of action of KSK-94.

## 1. Introduction

One of the causes of developing obesity is the consumption of more calories than the organism needs [1]. Due to their great palatability and lack of satiating capacity, meals with a sweet taste and high fat content are often overindulged by people [2]. Consequently, eating a high-fat meal impairs one’s capacity to cut back on food consumption [3,4] especially those both high in both fat and sugar [5].

In earlier studies, it was noted that histamine release was decreased by sweet substances such as sucrose and saccharin solutions. This could imply that appetizing food inhibits the release of histamine, which causes overeating [6]. Brain histamine acts as a relay station, combining central and peripheral information to regulate hunger and energy expenditure. It also affects the emotional significance of various experiences [7], including the hedonic value of food [8]. Therefore, the consumption of delicious meals and the subsequent development of obesity may be inhibited by an increase in histamine production and an intensification of the histaminergic signal by histamine H_3_ receptor antagonists.

The sigma-2 receptor remains an orphan receptor because endogenous ligands are still elusive. It is a very interesting target for potential drugs [9] and its physiological significance is still a puzzle with a therapeutically valuable solution in various disorders. Interestingly, the sigma-2 receptor (TMEM97) is involved in cholesterol homeostasis [10,11]; therefore, studies showing what changes may occur in obese organisms [12] or those at risk of developing obesity and treated with ligands of this receptor are important.

Chronic nutrient excess leads to the expansion and dysfunction of visceral adipose tissue (VAT) composed of unilocular cells of white adipose tissue (WAT). In addition to adipocytes, VAT includes multipotent stem cells, preadipocytes, fibroblasts, endothelial cells as well as immune system cells and the extracellular matrix, whose interactions decide the adipose tissue homeostasis [13]. During obesity, VAT undergoes remodeling, which includes not only an increase in the size and number of adipocytes [14] but also the infiltration of immune cells, death of adipocytes, and the accumulation of extracellular matrix components [13]. Adipocyte hypertrophy during VAT expansion is associated with the development of local hypoxia caused by limited blood supply and the restricted capacity of oxygen to diffuse in enlarged adipocytes [15,16]. Therefore, chronic hypoxia has been suggested to be part of pathogenic pathways leading to cellular stress, adipocyte death, inhibition of adiponectin expression, leptin hyperproduction, and inflammation [16,17,18,19]. Adipocyte death during obesity causes the accumulation of macrophages, lymphocytes, and other immune cells forming the so-called “crown-like structure”, a unique histological hallmark of obese adipose tissue [13,20]. Interactions between accumulated immune cells in VAT ultimately lead to increased secretion of pro-inflammatory cytokines such as macrophage migration inhibitory factor (MIF), monocyte chemoattractant protein (MCP)-1, tumor necrosis factor (TNF)-α, and interleukins (IL-6, IL-8, IL-10) [21]. Along with inflammatory cells, VAT adipocytes also secrete proinflammatory cytokines and other adipocytokines, whose secretion is altered in obesity-related diseases. The best-studied adipocytokines are leptin, adiponectin, and resistin. Leptin concentration is regulated by food intake and correlates with fat tissue mass, hence hyperleptinemia is observed in obesity [22]. Adiponectin improves glucose and lipid metabolism and prevents inflammation [23,24]. Resistin modulates insulin sensitivity, has pro-inflammatory properties, regulates glucose and lipid metabolism, as well as energy homeostasis by controlling food intake [25,26]. Modulating the pathways involved in these processes may provide new diagnostic and therapeutic opportunities in obesity treatment.

In addition to WAT, the adipose organ also consists of brown adipose tissue (BAT), formed by multilocular adipocytes. Two basic types of adipose tissue, white and brown, are described that differ in their origin, morphology, and function [27]. The classical theory states that brown adipose tissue is a thermogenic tissue that plays a critical role in regulating the body’s core temperature, while white adipose tissue mostly stores surplus energy in the form of triglycerides [28]. The brown adipocytes exist as the classical, or constitutive cells that arise during embryogenesis, and the bright (brown-in-white) or beige adipose tissue is recruited postnatally within white adipose tissue. It has also been found that brown adipocyte-like cells can be dispersed in white adipose tissue [27]. When stimulated by exercise, cold exposure, and other conditions, white adipocytes have considerable plasticity and can transdifferentiate into beige adipocytes, which share many morphological and functional traits with brown adipocytes. Another name for this occurrence is “browning of white adipose tissue”. Beige adipocytes can also arise through de novo differentiation from tissue-resident progenitors in addition to transdifferentiation. An innovative approach to enhancing the metabolism of glycolipids and reducing the need for insulin secretion in obese and type 2 diabetes patients may involve stimulating brown adipose tissue and causing the browning of white adipose tissue [28]. Moreover, the amount of brown adipose tissue positively correlates with energy expenditure and is significantly lower in obese persons than in slender individuals. Therefore, strategies aimed at the induction and/or activation of brown adipose tissue could be potentially useful in the treatment of obesity [27,28].

**Figure 1:**
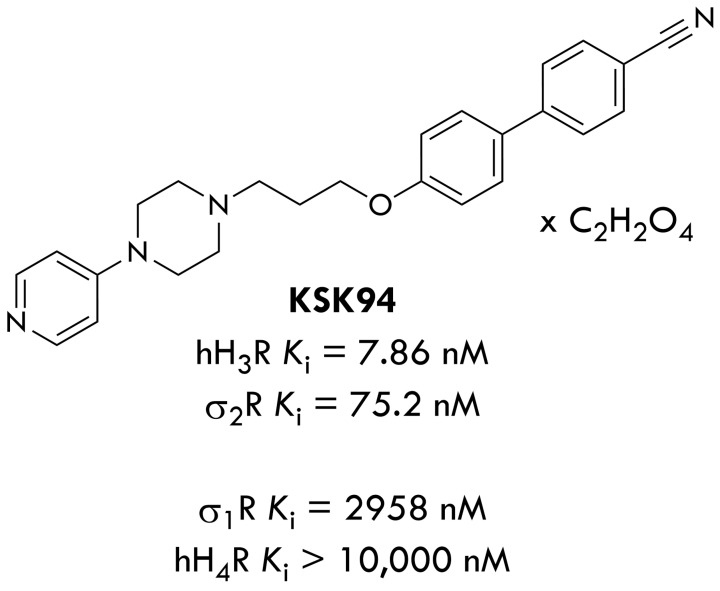
The structure of KSK-94 and affinity at the histamine H_3_ and sigma-2 receptors. The key interactions between the ligand and these two biological targets, explaining its high activity, have been described in our previous article [32].

Dual ligands of histamine H_3_ and sigma-2 receptors are very promising compounds in the reduction of body weight and selected metabolic disorders. Our previous studies showed a significant reduction in the development of obesity in rats in the excessive eating model when using various compounds, dual ligands, from the group of 4-pirydyl-piperazine derivatives [29,30,31]. We aimed in this research to investigate how a compound (KSK-94) with the strongest affinity at the histamine H_3_ receptor from this group, and significant binding to the sigma-2 receptor, which significantly reduced the development of obesity and selected metabolic disorders [32], influenced adipose tissue—the number and size of adipocytes and released factors (adipokines, factors involved in inflammation) in this model of developing obesity. For this purpose, we performed histopathological, immunohistochemical, and biochemical tests in adipose tissues collected from animals from our previous experiment. Figure 1 shows the structure of KSK-94 and affinity at the histamine H3 and sigma-2 receptors.

We picked bupropion and naltrexone as reference medications since they are commonly used to treat obesity [33]. The neurobiological mechanisms underlying the weight loss effects of the bupopion/naltrexone combination are not fully known, although they are thought to involve impacts on the reward system and diminish food cravings. Bupropion is licensed for use in depression and smoking cessation. It is considered to increase dopamine levels at specific brain regions, resulting in decreased hunger and increased energy expenditure. Naltrexone is approved to treat both opioid addiction and alcoholism. It may lower reward sensitivity to natural reinforcers and lessen reward for sweet-tasting foods [34].

## 2. Results

### 2.1. Assessment of the Development of Obesity and the Amount of Selected Hormones in Adipose Tissue

The final body weight in the palatable diet + vehicle group of rats was significantly higher as compared to control rats fed with only a standard diet after a four-week special diet. However, the final body weight was significantly lower following the treatment with KSK-94 or bupropion/naltrexone vs. palatable diet +vehicle group.

Similarly, palatable diet + vehicle-treated rats had significantly more visceral adiposity tissue than control animals. Treatment with KSK-94 or bupropion/naltrexone markedly prevented the growth of the amount of visceral fat (Table 1).

**Figure 2:**
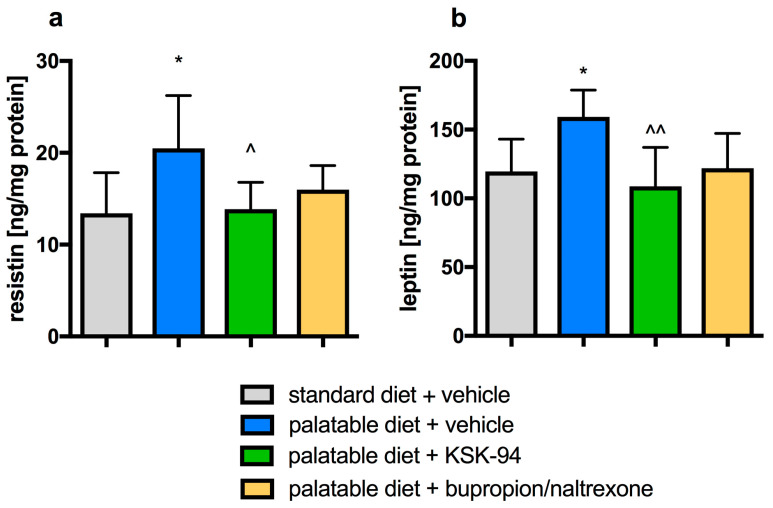
Effects of KSK-94 (10 mg/kg i.p.) or bupropion/naltrexone (20 + 1 mg/kg i.p.) on resistin (**a**) and leptin (**b**) concentration in visceral adipose tissue. Data are expressed as means ± 95% CI, (*n* = 6), * *p* < 0.05 vs. standard diet + vehicle, ^ *p* < 0.05, ^^ *p* < 0.01 vs. palatable diet + vehicle group (one-way ANOVA, post hoc Tukey multiple comparisons test).

Palatable diet consumption caused increased resistine level (by 52.7% vs. standard diet + vehicle group, *p* < 0.05, Figure 2a) and increased leptin level (by 33.3% vs. standard diet + vehicle group, *p* < 0.05, Figure 2b) in rats’ visceral adipose tissue. Treatment with KSK-94 prevented these changes. We observed a similar direction of effect with bupropion/naltrexone; however, it was without statistical significance (Figure 2).

### 2.2. Analysis of Adipocytes, Inflammatory Cells and Capillary Congestion

**Figure 3:**
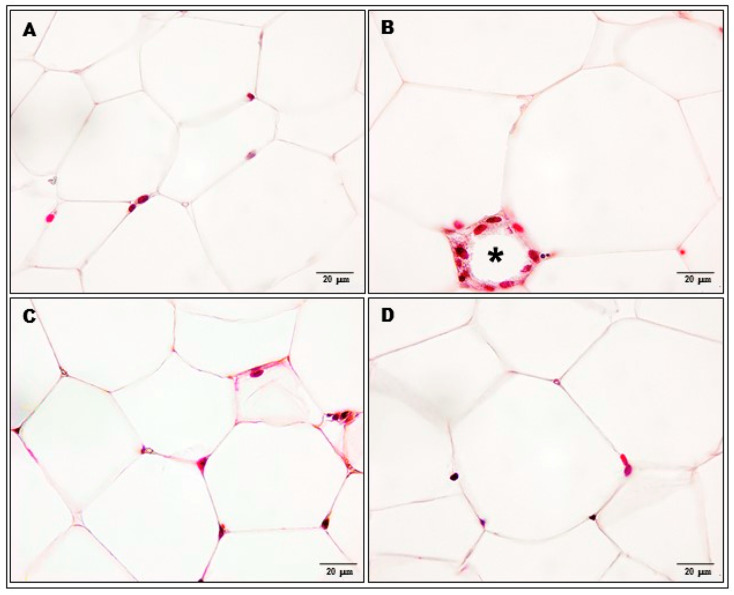
Decrease in adipocyte number in palatable diet + vehicle group (**B**) compared to both standard diet + vehicle rats (**A**) and palatable diet + KSK-94 rats (**C**). The mean number of adipocytes in the adipose tissue of the palatable diet plus bupropion/naltrexone-treated rats (**D**) was intermediate between the adipocyte number in standard diet + vehicle (**A**) and palatable diet + vehicle rats (**B**). Adipose tissue from palatable diet + vehicle rats (**B**) showed dead or dying adipocyte surrounded by macrophages forming a crown-like structure (asterisk). Masson’s trichrome staining, scale bars 20 μm.

**Figure 4:**
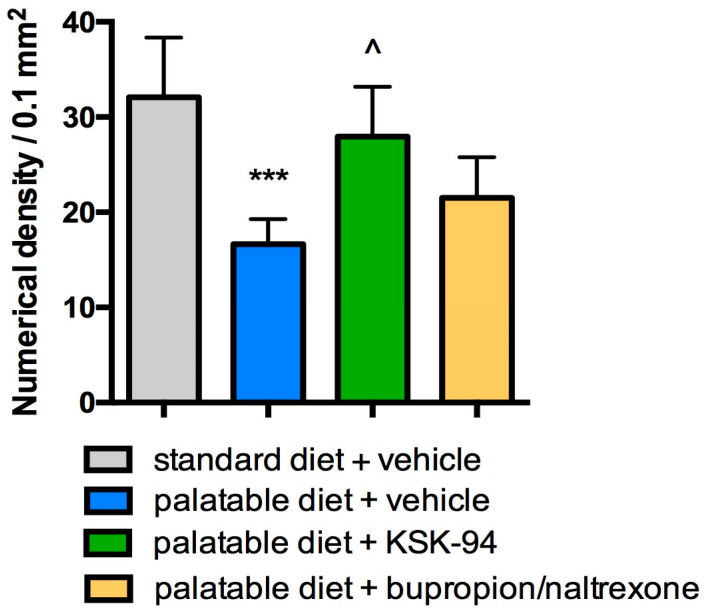
Numerical density of adipocytes from visceral adipose tissue of the different groups of rats. Data are expressed as means ± SD, *** *p* < 0.001 vs. standard diet + vehicle, ^ *p* < 0.05 vs. palatable diet + vehicle group (Kruskal–Wallis test by ranks, post hoc Dunn test).

Morphometric examination showed a statistically significant reduction in the number of adipocytes per 0.1 mm^2^ cross section area of adipose tissue in the palatable diet + vehicle compared to the standard diet + vehicle group (16.6 ± 1.1 vs. 32.1 ± 2.5, *p* < 0.001) but there was no reduction in the number of adipocytes in palatable diet+KSK-94 group. The number of adipocytes in the adipose tissue of these rats was 1.7-fold that of adipocytes in the adipose tissue from palatable diet + vehicle rats (27.9 ± 2.1 vs. 16.6 ± 1.1, *p* < 0.05) and did not differ statistically significantly from the density of adipocytes determined in the standard diet + vehicle group. The number of adipocytes in the adipose tissue of rats in the palatable diet + bupropion/naltrexone group showed intermediate values (21.5 ± 1.7) between the group of rats fed with a palatable diet + vehicle and the group with palatable diet + KSK-94. The differences were not statistically significant (Kruskal–Wallis test by ranks, post hoc Dunn test) (Figure 3 and Figure 4).

**Figure 5:**
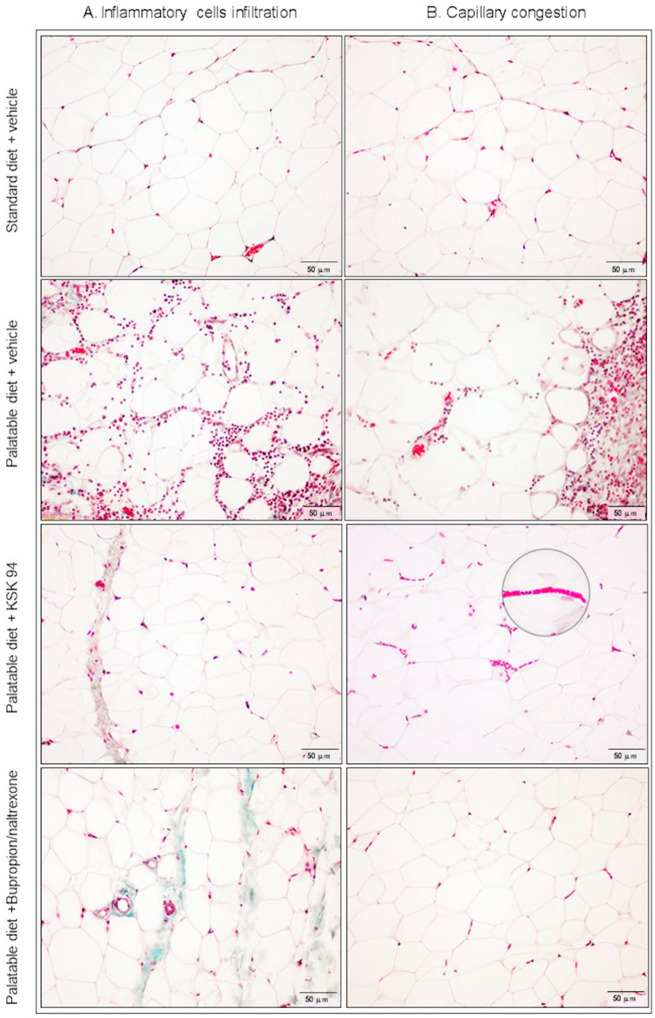
Representative histological pictures of adipose tissue sections from studied groups of rats stained with Masson’s trichrome, showing inflammatory cell infiltration (**A**) and capillary congestion (**B**). Numerous inflammatory cells were visible only in palatable diet + vehicle rats. In adipose tissue of palatable diet + KSK-94 rats, only slight inflammation was observed involving mostly septa, but most capillaries were heavily congested (in enlarged box). Scale bars 50 μm.

**Figure 6:**
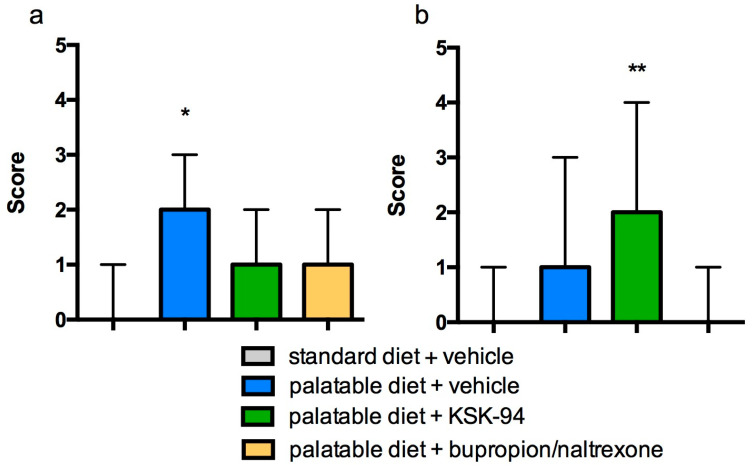
Inflammatory cell infiltration (**a**) and capillary congestion (**b**) in adipose tissue of the different groups of rats. (**a**). * *p* < 0.05 standard diet + vehicle versus palatable diet + vehicle. (**b**). * *p* < 0.05 palatable diet + KSK-94 versus palatable diet + bupropion/naltrexone and ** *p* < 0.01 standard diet + vehicle vs. palatable diet + KSK-94. Data are expressed as median + IQR, Kruskal–Wallis test by ranks, and post hoc Dunn test.

A similar trend also concerned the inflammatory response in adipose tissue. Heavily infiltrated with inflammatory cells (mononuclear cells and macrophages) was adipose tissue from palatable diet + vehicle rats. Moreover, we observed dead or dying adipocytes surrounded by macrophages forming crown-like structures (Figure 3B). A total of 66% of the rats from the palatable diet + vehicle group demonstrated an inflammatory cell infiltration judged as 2 or 3 grade (on a 0–3 scale, median-2, IQR-2) while standard diet + vehicle rats had only a small number of inflammatory cells in the fat tissue samples (0–1 grade, median-0, IQR-1) (Table 2). Statistical analysis of these findings showed a significant difference (*p* < 0.05). Whereas inflammatory cells were less common in the adipose tissue samples of palatable diet + KSK-94 (median-1, IQR-0) and palatable diet + bupropion/naltrexone rats (median-1, IQR-1) compared to palatable diet + vehicle tissue samples and infiltrated mostly septa of adipose tissue of these rats (Figure 5 and Figure 6a). However, no statistically significant differences were found between these groups. Also, a noteworthy observation was the common capillary congestion occurring in the adipose tissue of palatable diet+KSK-94 rats compared to all other groups. Statistically significant differences in capillary congestion of adipose tissue were found between palatable diet + KSK-94 rats versus standard diet + vehicle rats (*p* < 0.01) and palatable diet + bupropion/naltrexone rats (*p* < 0.05), respectively, (Figure 6b). All numeric histologic gradings for each rat in groups were presented in Table 2.

**Figure 7:**
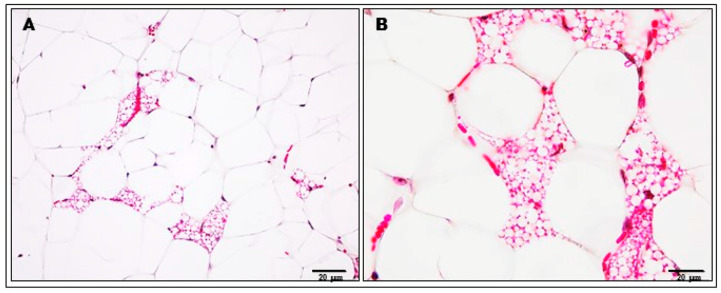
Histological pictures of visceral adipose tissue sections of palatable diet + KSK-94 group showing beige fat cells surrounded by white adipocytes. Masson’s trichrome stain. Scale bars: (**A**). 50 μm; (**B**). 20 μm.

**Figure 8:**
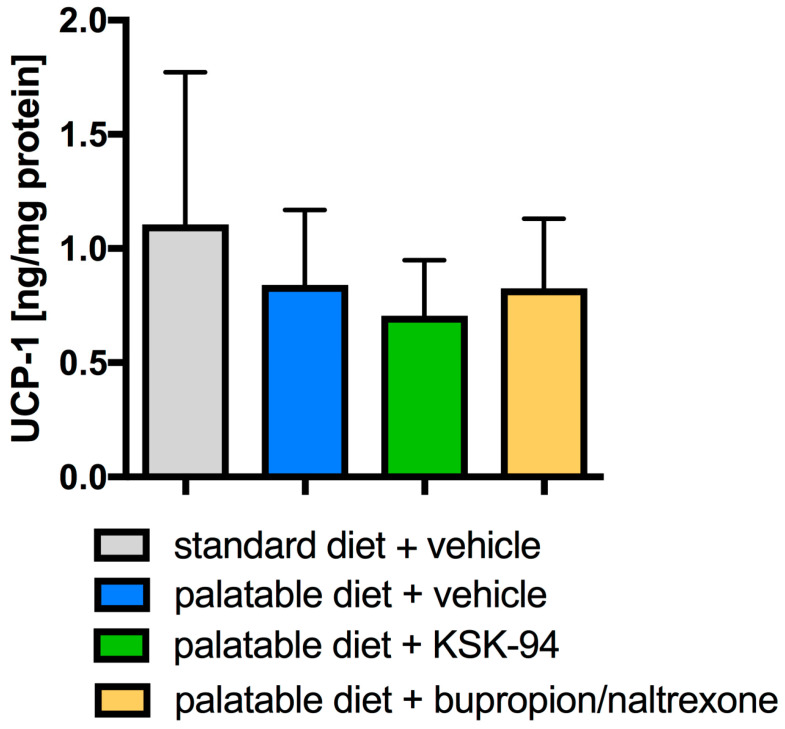
Effects of KSK-94 (10 mg/kg i.p.) and bupropion/naltrexone (20 + 1 mg/kg i.p.) on UCP-1 concentration in visceral adipose tissue. Data are expressed as means ± 95% CI, (*n* = 6), one-way ANOVA, post hoc Tukey multiple comparisons test.

A very interesting observation concerning only the adipose tissue of palatable diet animals treated with KSK-94 was the induction of browning of white visceral adipose tissue (Figure 7). As we noticed beige adipocytes possessing multiple lipid droplets, surrounded by unilocular white adipocytes, we studied the level of UCP-1 in adipose tissue; however, no statistically significant difference in UCP-1 concentration was found among groups (Figure 8). This observation requires further studies.

### 2.3. Evaluation of Inflammation in Adipose Tissue

**Figure 9:**
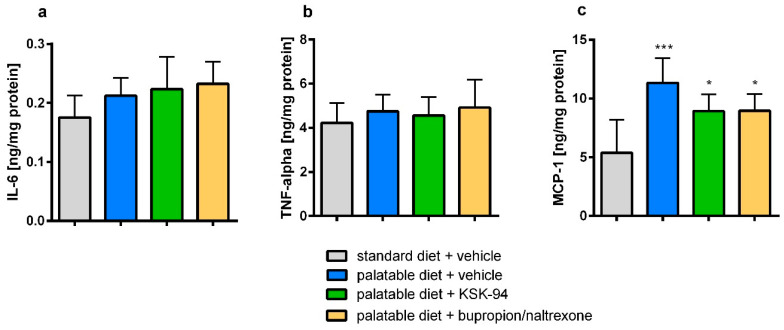
Effects of KSK-94 (10 mg/kg i.p.) and bupropion/naltrexone (20 + 1 mg/kg i.p.) on IL-6 (**a**) and TNF-a (**b**) and monocyte chemoattractant protein 1 (MCP-1, (**c**)) concentration in visceral adipose tissue. Data are expressed as means ± 95% CI, (*n* = 6), * *p* < 0.05, *** *p* < 0.001 vs. standard diet + vehicle group (one-way ANOVA, post hoc Tukey multiple comparisons test).

**Figure 10:**
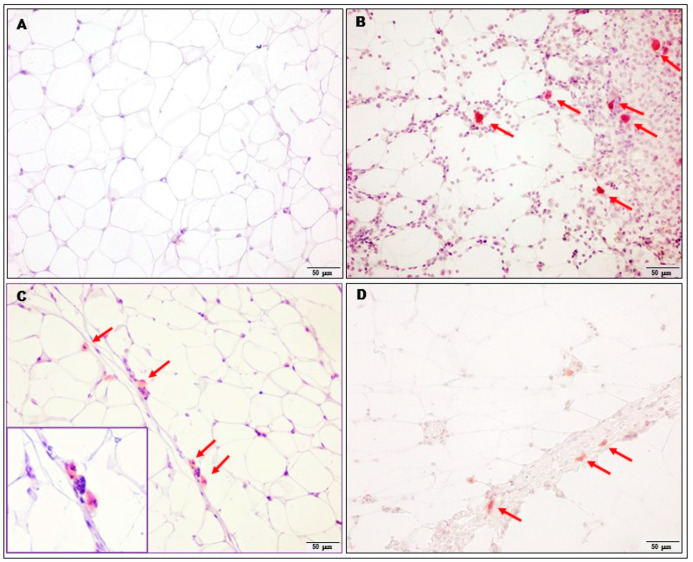
Immunoexpression of IL-6 was detected in some inflammatory cells (probably in macrophages, arrows, and enlarged box in (**C**) occurring more frequently in adipose tissue of palatable diet + vehicle rats (**B**). Only a few cells expressing IL-6 were observed in the adipose tissue of palatable diet+KSK-94 (**C**) and palatable diet + bupropion/naltrexone rats (**D**). There were no IL-6-positive cells in the adipose tissue of standard diet + vehicle rats (**A**). Immunohistochemical reaction counterstain with hematoxylin. Scale bars 50 μm.

There were no significant differences in the levels of interleukin-6 (IL-6) and tumor necrosis factor α (TNF-α) in adipose tissue between individual groups (Figure 9a,b). Many IL-6 positive cells (probably macrophages) were observed in fat tissue samples of the palatable diet + vehicle group, while in the other groups, they appeared sporadically (Figure 10).

Feeding with a palatable diet caused a statistically significant increase in monocyte chemoattractant protein 1 (MCP-1, by 110.3%, *p* < 0.001) concentration in visceral adipose tissue, compared with the values observed in rats on the standard diet. Treatment with KSK-94 or bupropion/naltrexone blocked raising this parameter but did not fully normalize elevated MCP-1 levels (Figure 9c).

**Figure 11:**
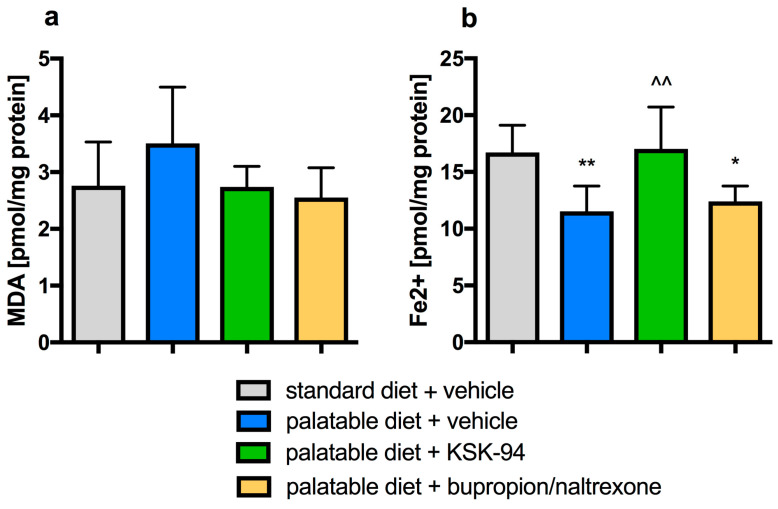
Effects of KSK-94 (10 mg/kg i.p.) and bupropion/naltrexone (20 +1 mg/kg i.p.) on MDA (**a**) and FRAP (**b**) concentration in visceral adipose tissue. Data are expressed as means ± 95% CI, (*n* = 6), * *p* < 0.05, ** *p* < 0.01 vs. standard diet + vehicle, ^^ *p* < 0.01 vs. palatable diet + vehicle group (one-way ANOVA, post hoc Tuckey multiple comparisons test).

Feeding with a palatable diet had no statistically significant effect on lipid peroxidation assessed in visceral adipose tissue homogenates by malondialdehyde levels (Figure 11). However, the total reduction capacity was reduced in obese animals, in adipose tissue. Treatment with compound KSK-94 significantly saved this reduction capacity, up to the level of capacity noted in tissues taken from control animals fed only with the standard diet. Such an effect was not observed after administration with bupropion/naltrexone.

## 3. Discussion

Our research was aimed at assessing adipose tissue after the treatment with a dual ligand of histamine H_3_ receptors and sigma-2 in rats in a model of developing obesity.

Our studies are preliminary studies, we do not show the exact connection between blocking the histamine H_3_ receptor and the effect on the sigma-2 receptor and blocking the development of all the unfavorable changes that we have observed in adipose tissue in rats from the control group with developing obesity. However, we clearly show the beneficial effects of the use of KSK-94, which is a selected representative of dual ligands of these receptors with a clear beneficial effect in preventing the development of obesity and significant adipose tissue disorders.

Adipose tissue can expand by hypertrophy, a rise in adipocyte size, hyperplasia, or an increase in adipocyte quantity brought on by the recruitment of additional cells during the development of obesity [14]. Adipocyte volume mirrors the balance of lipogenesis between lipolysis, while adipocyte number mirrors the ratio of preadipocyte proliferation, differentiation, and apoptosis of preadipocytes and the adipocyte [35]. When the body is given too many calories in the early stages of developing obesity, the number of adipocytes that secrete adipokines increases. Adipokines subsequently induce the production of more preadipocytes, which grow into mature adipocytes and shield the body from some of the harmful metabolic effects of obesity [36]. Our research clearly shows that in rats with developed obesity, adipocytes are larger and fewer in visceral adipose tissue. However, administration of the KSK-94 to rats significantly reduced the development of obesity and the size of adipocytes, while their number was clearly higher, similar to the number and size of adipocytes observed in the adipose tissues of rats from the control group consuming only standard feed. The compound we tested had a better effect in this respect compared to bupropion/naltrexone.

In addition to producing steroid hormones, white adipose tissue secretes a variety of peptide hormones, such as leptin, resistin, adiponectin, and many cytokines, as well as acylation-stimulating protein and adipsin, angiotensinogen, and plasminogen activator inhibitor-1, etc. It is not only an energy storage tissue but a significant endocrine organ that affects energy balance, vascular homeostasis, glucose and lipid metabolism, and immunological response [37]. The low-grade chronic inflammation, which is present in obesity, impacts the physiology of local adipose tissue and negatively impacts other tissues systemically [38]. This is primarily due to macrophage infiltration as well as increased secretion of many pro-inflammatory and decreased secretion of anti-inflammatory substances, like hormones (leptin), adipokines (resistin, adiponectin), and cyto- and chemokines (i.e., MCP-1, TNF-α, and IL-6) [39,40]. Our research shows that the administration of the KSK-94 to rats during unlimited availability of a diet that quickly induces obesity did not allow the development of inflammation and prevented the increase in leptin and resistin, saved the fat tissue reducing activity, which shows that the use of this compound contributed significantly to maintaining the proper functioning of adipose tissue in the body.

Long-term control of food intake, body weight, energy expenditure, and neuroendocrine function depend heavily on leptin. That adipose leptin production is increased in obese individuals, has led to the hypothesis of obesity-induced leptin resistance [37]. Our previous research showed that the administration of KSK-94 during unlimited availability of a palatable diet resulted in animals not consuming as many excess calories as animals from the control group with developing obesity [32]. This anorexic effect observed during the administration of KSK-94, which was very likely related to the effect of this compound on histamine H_3_ receptors and the intensity of histamine action, because this is a mechanism of action of this compound, may be related to the lack of increase in leptin levels in rats treated with KSK-94, observed in the present study. Through the activation of histamine H_1_ receptors, histamine functions as an anorexigenic agent. Histamine functions as a mediator between leptin’s appetite-inhibiting action and appetite regulation. Histamine may mitigate and postpone the onset of leptin resistance in obesity caused by a high-fat diet [41]. KSK-94 maintained leptin levels sufficient to allow for the proper functioning of the hunger and satiety centers. In this study, leptin levels were in direct correlation with levels of adipose tissue.

In the state of excess adipose tissue, elevated levels of resistin are also observed. This hormone influences some of the insulin target organs such as adipocytes, skeletal muscle cells, and the liver, and is a potential link between obesity and type 2 diabetes mellitus. Moreover, it has a direct effect on adipose tissue, manifested by the regulation of adipocyte proliferation and differentiation. This protein also participates in the regulation of energy homeostasis by controlling food intake and is considered a strongly pro-inflammatory compound [42,43,44]. Recent reports suggest that resistin is most likely involved in the development of leptin resistance [44]. An abnormal increase of resistin in obese subjects promotes free fat acid release, and increased lipolysis may contribute to the onset of insulin resistance [42,43]. In this study, we observed a significant increase in the level of resistin in the adipose tissue of obese animals, it is in line with literature data [45]. Additionally, we observed the development of inflammation, and although we did not determine a significant increase in the levels of cytokines such as IL-6 and TNF in adipose tissue, our studies showed a significant increase in MCP-1 in the adipose tissue of obese animals and a correlation between the level of resistin in adipose tissue and inflammation. Undoubtedly, a limitation of this work is the lack of plasma cytokine testing. Our results therefore show not only a reduction in the development of obesity and inflammation but also an increase in both leptin and resistin levels, and leptin resistance when using KSK-94 in animals with unlimited access to tasty food. The tested compound performed better in all tests compared to the reference bupropion/naltrexone. All these data form a logical whole of the beneficial effect of the administered compound in preventing the development of obesity and related complications in adipose tissue and the entire body.

Considering this study, an interesting observation is the ability of the KSK-94 to induce browning of visceral white adipose tissue. We observed beige adipocytes, multilocular cells, lying among the unilocular white adipose tissue cells of animals treated with this compound. Beige adipocytes, which appeared within white fat deposits following a variety of external stimuli, are an inducible form of thermogenic fat cells [46,47]. Adaptive thermogenesis refers to the body’s production of heat in response to external stimuli and is currently considered a potential new therapeutic approach to obesity. Numerous studies have shown that selective induction of beige adipocytes by genetic and pharmacological methods significantly increases the energy expenditure of the entire body and protects animals against diet-related obesity [48,49].

To confirm histological observations regarding the formation of beige adipocytes in the analyzed visceral adipose tissue, the concentration of the mitochondrial protein UCP-1 responsible for thermogenesis in brown adipose tissue cells was examined. The analysis did not show any differences in the concentration of this protein between the study groups. However, what attracted attention was the capillary congestion of adipose tissue, appearing especially in the areas where multilocular cells were present. Brown adipose tissue is more vascular than white adipose tissue because a significant blood supply is required to activate thermogenesis, which will provide the appropriate amount of oxygen and nutrients [50]. Despite the lack of differences in UCP-1 protein concentration between the analyzed groups, the possibility of browning of white adipose tissue in the group of animals treated with KSK-94 cannot be completely ruled out, because, in addition to UCP-1-dependent thermogenic mechanisms, beige adipocytes may also use recently described UCP-1-independent mechanisms [51,52,53]. The above observations, which are so important from a therapeutic point of view, require further studies.

In conclusion, the results of our research clearly indicate that animals administered with the tested compound did not develop obesity to the same extent as in the control group with obesity. KSK-94 prevented an increase in fatty tissue in the visceral cavity. The levels of determined hormones in the adipose tissue of animals from the palatable + KSK-94 group, such as leptin and resistin, after treatment, were at a comparable level as in the adipose tissue collected from the control group without obesity. The results are extremely interesting because we discovered that in addition to reducing adipocyte size and inhibiting local inflammation to some extent in adipose tissue, the compound KSK-94 can induce of browning of white adipose tissue.

Our study suggests that dual compounds with a receptor profile like KSK-94, i.e., targeting histamine H_3_ receptor and, to a lesser extent, sigma-2 receptor, could be attractive therapeutic options for patients at risk of developing obesity or with obesity and some metabolic disorders. However, more studies are required to determine its safety profile and the exact mechanism of action of KSK-94. Such information would make it possible to target the synthesis of new, effective compounds with anorexic activity, and provide metabolic benefits and potential use in preventing the development or alleviating the existing obesity.

## 4. Materials and Methods

### 4.1. Drugs and Chemicals

Compound KSK-94 was synthesized in the Department of Technology and Biotechnology of Drugs, Faculty of Pharmacy, Jagiellonian University [32]. Heparin was delivered by Polfa Warszawa S.A. (Warsaw, Poland), thiopental sodium was obtained from Sandoz GmbH, (Kundl, Austria), bupropion from TCI Co. (Fukaya, Japan), and naltrexone from Sigma-Aldrich (Munich, Germany).

### 4.2. The Experimental Protocol

The studies were performed on visceral adipose tissue from female rats fed with a palatable diet (Western-style diet, a model of excessive eating) collected in our previous experiment [32]. All experimental procedures were conducted in accordance with the guidelines of the Animal Use and Care Committee of the Jagiellonian University and approved by the Local Ethics Committee on Animal Experimentation (resolution no. 185/2017, 232/2019) in Kraków, Poland.

The model of excessive eating in rats, proposed in our study, perfectly illustrates the harmful high-caloric intake associated with overeating of freely available tasty products rich in sugar and fat [54,55]. In the model of excessive eating, animals are given access to high-caloric foods, as well as standard feed. Obesity is developing quickly because these animals want to consume palatable feed containing more calories than they need. The feeding is not forced in any way, rats are allowed to decide when, what, and how much to eat.

Female Wistar rats (Krf: (WI) WU), age 7 weeks, obtained from an accredited animal house at the Faculty of Pharmacy, Jagiellonian University Medical College, Kraków, Poland, were used.

Rats were randomly divided into 4 groups (*n* = 6) and studied for 4 weeks. The groups were as follows:

Control group. Animals were fed a standard diet (Labofeed B, Morawski Manufacturer Feed, Zdynia, Poland) water ad libitum, and received vehicle (1% Tween 80, 1 mL/kg i.p., daily) for 4 weeks.

Palatable control group (palatable diet + vehicle): Animals were fed a palatable diet with additional access to a standard diet (Labofeed B, Morawski Manufacturer Feed, Poland), water ad libitum and received vehicle (1% Tween 80, 1 mL/kg, i.p., daily) for 4 weeks.

Palatable diet + KSK-94 group: Animals were fed a palatable diet with additional access to a standard diet (Labofeed B, Morawski Manufacturer Feed, Poland), water ad libitum, and received KSK-94 (10 mg/kg, suspended in1% Tween 80, i.p., daily) for 4 weeks.

Palatable diet + bupropion/naltrexone group: Animals were fed palatable diet with additional access to a standard diet (Labofeed B, Morawski Manufacturer Feed, Poland), water ad libitum, and received bupropion/naltrexone (mixture, 20 and 1 mg/kg, respectively, suspended in 1% Tween 80, i.p., daily) for 4 weeks.

**Figure 12:**
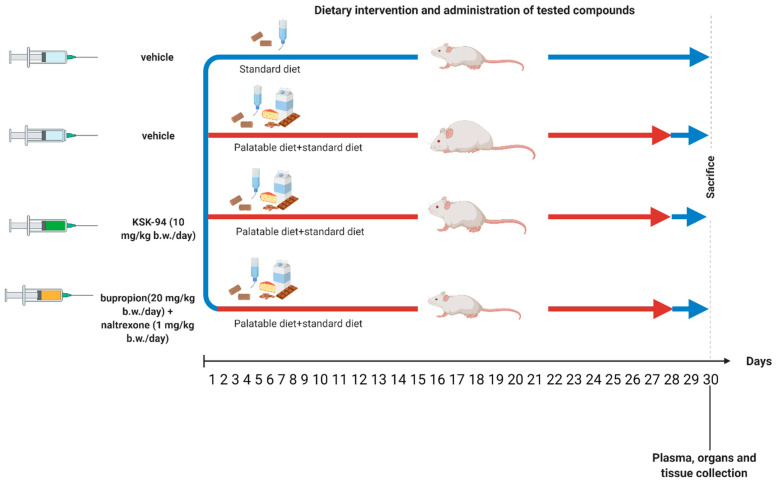
Timeline of the experiment.

For four weeks, rats given a palatable meal had access to both a regular feed (Labofeed B, Morawski Manufacturer Feed, Poland) and a milk chocolate diet with nuts, cheese, salted peanuts, and 7% condensed milk [29,30,31]. The water was available ad libitum. The animals were provided with standard feed in addition to palatable products. Figure 12 shows the timeline of the experiment.

At the end of the experiment, after 16 h fasting but with free access to water, all rats were anesthetized with thiopental (75 mg/kg i.p.), decapitated, and visceral adipose tissue was dissected out and weighed by investigators unaware of the group’s allocation. Some part of the collected adipose tissue was frozen and stored at −80 °C until assayed (less than 3 months) and some were fixed in 4% formaldehyde for histopathological analysis.

The palatable diet contained: milk chocolate with hazelnuts—100 g, 195 kcal; condensed milk—100 mL, 131 kcal; peanuts—100 g, 614 kcal; cheese (Greek type)—100 g, 270 kcal. A standard diet provided 280 kcal/100 g of feed.

### 4.3. Histological Examination

Adipose tissue samples were fixed in 4% formaldehyde, embedded in paraffin, and cut into 5 μm thick sections for either Masson’s trichrome staining or immunohistochemical analysis (IL-6 expression). Six random fields from each section were analyzed in a blinded manner with an Olympus BX41 light microscope (Olympus BX41, Evident, Tokyo, Japan) using a 20X, 40X, and 100X objective, and digital images were captured with an color camera (Olympus UC90, Evident, Tokyo, Japan). For quantitation of the number of adipocytes, eight fields/slides under 100X objective were analyzed using an image analysis system CellSensDimension v. 1.18 (Olympus, Tokyo, Japan). The numerical density of adipocytes was calculated by counting the total number of adipocytes per high-power field (31,324 μm^2^) and then this number was converted to 0.1 mm^2^. According to stereometric rules, adipocytes tangent to the left and top edge of the image field were not counted. The presence of infiltrated immune cells and capillary congestion were graded either on a semiquantitative 4-point scale ranging from 0 to 3+ (0—minimal, 1—mild, 2—moderate, 3—severe) or on a semiquantitative 3-point scale ranging from 0 to 2+ (0—minimal, 1—moderate, 2—severe), respectively.

### 4.4. Immunohistochemistry

For immunohistochemistry, 5 μm serial sections of adipose tissue were dewaxed, re-hydrated, and treated for heat-mediated antigen retrieval (Cell Marque, Rocklin, CA, USA). Endogenous peroxidase activity was quenched with 3% H_2_O_2_/methanol for 15 min and tissue sections were blocked with normal goat serum (MILLIPORE, Burlington, MA, USA) for 10 min at room temperature to reduce nonspecific staining. Next, serial sections were incubated overnight (4 °C) with 0.125 g/mL of a mouse anti-rat monoclonal IL-6 primary antibody (ab9324, Abcam, Cambridge, UK). Slides that received no primary antibody served as negative controls. After several washes in TBS, the sections were incubated with a biotinylated secondary IgG, then with streptavidin-biotin horseradish peroxidase complex for 10 min in accordance with the instructions from the producers (IHC Select Immunoperoxidase Secondary Detection System, (MILLIPORE, Burlington, MA, USA)) and were developed using 3,3′-diaminobenzidine (DAB, MILLIPORE, Burlington, MA, USA) for 6 min. Each section was counterstained with hematoxylin. Adipose tissue slides with the expressing IL-6 were evaluated using an optical microscope coupled to a digital camera using a 40X objective.

### 4.5. Preparation of Tissue Homogenates

The frozen visceral adipose tissue was weighed, and homogenates were made by homogenizing 1 g of the tissue in 4 mL of 0.1 M phosphate buffer, pH 7.4, using the IKA-ULTRA-TURRAX T8 homogenizer (IKA Poland Sp. z o.o., Warszawa, Polska). Adipose tissue homogenates were next used for biochemical assays.

### 4.6. Biochemical Assays

The concentrations of IL-6 (E0135Ra, Bioassay Technology Laboratory, Shanghai, China), TNF-α (E0764Ra, Bioassay Technology Laboratory, Shanghai, China), MCP-1 (E0193Ra, Bioassay Technology Laboratory, Shanghai, China), UCP-1 (E2153Ra, Bioassay Technology Laboratory, Shanghai, China), resistin (E0211Ra, Bioassay Technology Laboratory, Shanghai, China), leptin (E0561Ra, Bioassay Technology Laboratory, Shanghai, China) in adipose tissue were determined by the ELISA method with the use of commercially available kits, according to the manufacturer’s protocol. To determine the levels of protein, a standard spectrophotometric test (Cat. No 1-008-B210, Biomaxima S.A. Lublin, Poland) was used.

Analyte levels were determined based on standard curves using the curve-fitting method and expressed in pmol/mg protein or ng/mg protein.

### 4.7. Determination of Lipid Peroxidation (Malondialdehyde Level)

The spectrophotometric assay, which was previously published, was used to measure the quantity of malondialdehyde (MDA), an indication of lipid peroxidation [56,57]. Briefly, 250 µL of a tissue homogenate was added to 250 µL of distilled water, 500 µL of 15% TCA, and 500 µL of 0.37% TBA. The samples were heated in a boiling water bath for 10 min. After cooling, the samples were centrifuged at 12,000× *g* for 10 min. The absorbance of the supernatant was measured at 535 nm.

### 4.8. Ferric-Reducing Antioxidant Power (FRAP) Assay

The assay was carried out according to Benzie and Strain [58] with some modifications [59,60]. The FRAP working solution was prepared before the start of the analysis: 0.3 mol acetate buffer (pH 3.6), 0.01 mol TPTZ (2,4,6-tripyridyl-s-triazine; Sigma-Aldrich) in 0.04 mol HCl (POCh, Poland) and 0.02M FeCl_3_ × 6H_2_O in water (iron (III) chloride hexahydrate; Chempur, Poland) were mixed in a volumetric ratio of 10:1:1 and protected from light. Next, 20 µL of adipose tissue homogenate was mixed with 180 µL of the FRAP working solution. The mixtures obtained were incubated at 37 °C for 30 min and their absorbance was measured at 593 nm. FeSO_4_ × 7H_2_O (100–1000 µM/L) was used for a calibration curve. Deionized water with FRAP solution was used as a blank.

### 4.9. Statistical Analysis

The results are expressed as means ± 95% of confidence interval (CI) or standard deviation (SD). Statistically significant differences between groups were determined using one-way ANOVA and the post-hoc Tukey multiple comparison test, which included all feasible pairwise comparisons. Histologic rank scores are expressed by median and inter-quartile range (IQR). The comparisons among groups for all histopathological studies were performed using the Kruskal–Wallis test by ranks followed by Dunn’s post-hoc test with all pairwise comparisons. Differences were considered significant at *p* < 0.05.

## Figures and Tables

**Table 1:** Body and visceral fat weight.

Group	Initial Body Weight (g)	Final Body Weight (g)	Visceral Fat Weight (g)
standard diet + vehicle	187.8 ± 4.9	218.8 ± 7.5	4.2 ± 1.1
palatable diet + vehicle	190.8 ± 2.0	298.2 ± 19.7 ***	8.9 ± 2.6 ***
palatable diet + KSK-94	193.4 ± 1.9	253.9 ± 10.6 ^##^	2.6 ± 0.4 ^###^
palatable diet + bupropion/naltrexone	192.9 ± 5.7	265.7 ± 17.1 ^##^	4.2 ± 0.4 ^###^

Values are presented as mean ± SD, *n* = 6. *** *p* < 0.001 vs. standard diet + vehicle, ^##^ *p* < 0.01, ^###^ *p* < 0.001 vs. palatable diet + vehicle, one-way ANOVA, post hoc Tukey multiple comparison test.

**Table 2:** Histologic scores in adipose tissues of studied groups. Number of rats within groups with appropriate histologic grade.

Grade	Inflammatory Cells Infiltration				Capillary Congestion		
	Minimal	Mild	Moderate	Severe	Minimal	Moderate	Severe
standard diet + vehicle	4	2	-	-	5	1	-
palatable diet + vehicle	-	2	2	2	1	4	1
palatable diet+KSK-94	1	4	1	-	-	1	5
palatable diet + bupropion/naltrexone	1	3	2	-	4	2	-

Histologic rank scores are expressed by median.

## Data Availability

The data described in this study are accessible upon request.
